# Severity scores and their associated factors among orally poisoned toddlers: a cross sectional single poison center study

**DOI:** 10.1186/s40360-015-0044-7

**Published:** 2016-01-04

**Authors:** Menyfah Q. Alanazi, Majed Al-Jeraisy, Mahmoud Salam

**Affiliations:** Drug Policy and Economic Center, Riyadh, Saudi Arabia; King Abdullah International Medical Research Center (KAIMRC), Riyadh, Saudi Arabia; Pharmaceutical care, King Saud bin Abdulaziz University for Health Sciences (KSAU-HS), Riyadh, Saudi Arabia; National Biobanking Section, King Abdullah International Medical Research Center (Mail Code 1515), Ministry of National Guard Health Affairs, Riyadh, 22490 Saudi Arabia

**Keywords:** Toddlers, ingestions, poison, severity score, factors

## Abstract

**Background:**

One of the most unfortunate events toddlers may encounter during their early years of curiosity and experimentation is substance poisoning. The aim of the study was to evaluate the poison severity score and its associated factors among toddlers with orally ingested substances at a pediatrics emergency department (ED), central Saudi Arabia.

**Methods:**

A cross-sectional, poisoning report review between 2009&2011 was conducted. Exposures were patient characteristics (sex, age, body mass index, medical history) and incident characteristics (substance type, amount, form, witnessed or not, home remedy, arrival time to ED). Outcome was Poison Severity Score (PSS) that rates signs/symptoms of 11 body aspects on scale 0–4 (none, minor, moderate, severe, fatal). Inclusion criteria: age (1–3 years), previously healthy and oral exposure route. Bivariate analysis and multi-linear regression were conducted. Significance at p < 0.05.

**Results:**

Eligible cases were 165/315(52 %). Males (58 %) and females (42 %) had normal BMI (70 %). Substances ingested were medications (60 %) and chemicals (40 %). Almost 85 % were witnessed incidents and 27 % received a home remedy (water, juices, dairy products, salt/sugar solutes, and/or manually induced vomiting). Delayed arrival (≥1 hour) was observed in 57 %. Composite mean PSS of total was (0.16 ± 0.21), and was highest at the gastrointestinal (GI) aspect (0.39 ± 0.63), metabolic balance (0.35 ± 0.60), and respiratory aspect (0.30 ± 0.61). Significantly associated factors with higher severity scores were: home remedies at the composite mean PSS (adj.p = 0.048), chemical poisoning at two aspects respiratory (adj.p = 0.047) and muscular (adj.p = 0.009) compared to medication poisoning. Unwitnessed incidents at the muscular aspect (adj.p = 0.026) compared to witnessed incidents; delayed arrival time to ED at three aspects GI (adj.p = 0.001), nervous system (adj.p = 0.014) and kidney (adj.p < 0.001).

**Conclusions:**

Parents are not recommended to provide any home remedy to their orally poisoned toddlers, but rather directly visit the ED. Physicians are expected to observe more severe clinical outcomes among toddlers with chemical poisoning, unwitnessed incidents, and delayed arrival times especially at the respiratory, GI, muscular, nervous and kidney aspects.

## Background

One of the most unfortunate events toddlers may encounter during their early years of curiosity and experimentation is substance poisoning [1, 2]. Poison control centers in the United States received more than 2.4 million reports in 2003, of which 45.7 % were aged ≤ 3 years [3]. Pooling of millions of poison reports among children by the American Academy of Pediatrics (AAP) has generated solid evidence based recommendations and management guidelines, which dramatically decreased such unfortunate events over the years [4]. In Saudi Arabia, one poison center in the Western region noted that between 2008–2012, 57/129 (44 %) of poisoned children aged less than 12 years [5], while 1,272 poisoned children (1–15 years) were identified by the Eastern regional poison center between 2011-2013 [6].

Poison management in hospitals is based on an appropriate supportive and/or toxic-specific treatment [7–11]. At homes, Ipecac (a 1-oz bottle of over the counter syrup) had been recommended as a safe emetic between 1965 and 2003 [12]. However, the American Academy of Clinical Toxicology clearly stated that Ipecac should no longer be used due to its lack of efficacy [4, 7, 13], yet it is still enlisted on the Saudi Food and Drug Authority (SFDA) consumer awareness articles as of 2013. Activated charcoal usage dates back further as a traditional gastric decontaminant [14], yet its routine usage is discouraged, especially after one hour of substance ingestion [8, 15]. In addition, some guidelines recommended dilution by drinking 100 to 200 mL of water, but only for chemical substance ingestions [4].

In general poison center experts discourage any sort of home poison management and advise parents to notify poison centers or visit the emergency department (ED) for professional management [16–18]. To our knowledge home remedies for poison incidents do exist in our local community, but was not attended to clearly in previous studies [18] , especially among the high risk group of toddlers [2, 3, 15, 19].

Aim was to evaluate the poison severity score (PSS) of toddlers complaining of orally ingested substances at a single poison center, of a tertiary care facility, central Saudi Arabia. This was fulfilled by: 1) Obtaining the characteristics of toddlers and poisoning incident, 2) Evaluating their Poison Severity Scores (PSS), 3) Identifying significantly associated factors with high PSS.

## Methods

### Study design

This is a retrospective cross sectional, review of poisoning reports conducted at King Abdulaziz Medical City (KAMC). KAMC is a distinguished Joint Commission International (JCI) accredited tertiary health care facility established in 1983. KAMC is a certified poison center enlisted under the National Drug & Poison Information Center (NDPIC) and responds to any public or health care professional queries regarding any poison incident.

Within the vicinity of KAMC, a pediatric ED has an estimate of 85 beds allocated for pediatrics admitted and requiring various emergency care levels. The ED has a team of more than 70 emergency specialized pediatric medical staff who provide services to numerous admissions annually [20]. On call toxicologists or physicians with advanced training on toxicology are readily available at all times.

**Fig. 1:**
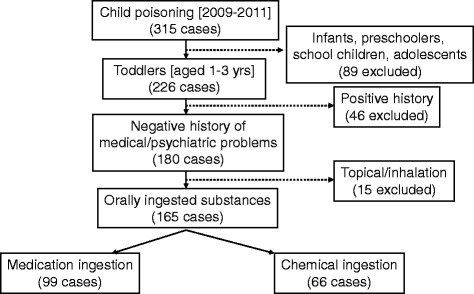
Inclusion/exclusion criteria

Poisoning reports of children complaining of acute poisoning (medication and/or chemical substance) between 2009 and 2011 were reviewed. Inclusion and exclusion criteria are illustrated in Fig. 1. The age was restricted to the high risk group of toddlers (1-3years). Any case with a previous health condition, such as asthma, was excluded to control for any potential health confounder that may result in a more complicated or deteriorated PSS. Cases of intentional over-dosage or suspected domestic violence were excluded too.

### Data collection

Poisoned children were triaged and attained for by licensed ED physicians. As per hospital policy, a drug/chemical poisoning form needs to be filled and signed by the medical staff based on an their initial clinical assessment. Study investigators incorporated their data collection forms with the hospital reporting forms (between 2009–2011) based on an agreement with the chairman of department. This agreement was supported by a research scientific committee, ethics committee and chief executive office approval memorandums. Data collection forms were stored in patients’ charts.

Study investigators delivered group training sessions for a team of 35 ED pediatric physicians on how to properly obtain the informed consent from parents or legal guardians, gather study related information and grade the observed clinical signs/symptoms on an evaluation tool. Two certified clinical research coordinators from King Abdullah International Medical Research Center (KAIMRC) were also assigned and trained to follow-up daily on this process and ensure the forms are properly completed. Non-eligible patients or those with unclear contact information were dropped out. Missing items in the forms were reported to study investigators and dealt with statistically. Validation of the data collected was done by verifying it with the medical records and by phone calling the parents (1–2 days after the incident). Phone calls after discharge were very important as questioning the anxious and stressed parents during the initial ED visit often leads to an inaccurate history or description of the incident details [12, 21].

The data collection forms were comprised of:

1. Informed consent: names, medical record number, date/time, contact information, signatures.
2. Toddler characteristics: age (1–3 years), sex, medical/psychiatric history, body mass index (BMI) for 2–3 year old toddlers, plotted on sex-specific growth charts [22],and classified as under weight (<5^th^ percentile), normal weight (5-85^th^ percentile), over weight (86^th^ – 94^th^ percentile), and obese (≥95^th^ percentile).
3. Poison incident characteristics: substance type (medication,chemical), number of agents, estimated amount, form (pill,capsule,liquid,cream), exposure route, witnessed or not, poison center informed or not, arrival time to ED (hours), home remedies provided. This section was sourced out from the reporting forms used by the Saudi Ministry of Health (MOH) and NDPIC.
4. Outcome characteristics: Initial clinical assessment (upon arrival) was conducted qualitatively using the Poison Severity Score (PSS) [23], a standard tool for grading the severity of poisoning. The PSS takes into account the overall clinical picture and is applicable to both subjective symptoms and objective signs. PSS uses a 5-level grading system ranging from no symptoms (zero), mild, transient and spontaneous resolve of symptoms/signs (one), pronounced or prolonged symptoms/signs (two), severe or life threatening symptoms/signs (three), and fatal (four). PSS assesses and grades 12 body aspects which are the GI- tract, Respiratory tract, Nervous system, Cardiovascular, Metabolic balance, Liver, Kidney, Blood, Muscular system, Local effects on skin, Local effects on eye and Local effect of Bites/Stings (not applicable in this study).

### Ethical considerations

All study personnel preserved the confidentially of the patients’ information as part of their job requirements. Patient identifiers were recorded to follow-up on the outcomes and validate the data collected. Signed informed consents were obtained from the parent or legal guardian of study participant, stapled to the data collection forms and preserved in patients’ charts. Study investigator had no influence on parents self-reporting. This study was approved by the Institutional Review Board of the Ministry of National Guard Health Affairs (MNG-HA), Riyadh, Saudi Arabia (RR08/019).

### Data analysis

SPSS statistical software (Version 22; SPSS Inc., Chicago, IL, USA) was used for data entry and analysis. Categorical variables such as sex, BMI group, and others were presented in frequency and percentage, whereas continuous variables such as PSS scores were presented in mean (x), standard deviation (±SD), and 95 % confidence interval (95 % CI). Individual aspects of PSS were graded 1^st^ qualitatively (categorical) and quantitatively (mean scores). Composite mean score of PSS for each toddler was calculated by the score summation of 11 aspects (12^th^ excluded) divided by 11. Bi-variate analysis was conducted using student t-test and one way ANOVA. Multilinear regression was constructed to identify the significant associations with higher PSS and control for all possible confounders. Significance level was initially set at P-value < 0.05 and after applying the Bonferroni correction, the corrected P-value was found significant at <0.049.

## Results

Study subjects who meet the eligibility criteria were 165/316 (52 %), Fig. 1. Male toddlers were at higher risk (58 %) compared to females (42 %), but with no significant gender differences (p = 0.606). BMI showed that the majority had normal weight 70.2 %, while underweight were 4.4 %, and overweight to obese 25.4 %, with no significant differences between these weight groups, p = 0.569, Table 1.

**Table 1:** Toddler and poison incident characteristics compared by the composite mean of Poison Severity Score

	Frequency - n (%)	Poison Severity Score - x ± SD	95 % CI
	165 (100.0)	0.16 ± 0.21	(0.13-0.19)
Sex			
Male	96 (58.2)	0.18 ± 0.23	(0.13-0.23)
Female	69 (41.8)	0.16 ± 0.16	(0.12-0.20)
		t = 0.554, p = 0.580	
Age of the toddler			
1–2 years	119 (72.1)	0.18 ± 0.22	(0.14-0.22)
2.-3 years	46 (27.9)	0.14 ± 0.16	(0.09-0.19)
		t = 1.021, p = 0.309	
BMI Percentile			
Underweight (<5^th^)	5 (4.4)	0.13 ± 0.14	(0.01-0.25)
Normal weight (5^th^-85^th^)	80 (70.2)	0.17 ± 0.21	(0.12-0.22)
Over weight (86^th^ – 94^th^)	14 (12.3)	0.10 ± 0.16	(0.02-0.18)
Obese (≥95^th)^	15 (13.1)	0.15 ± 0.15	(0.07-0.23)
		F = 0.657, df = 3, p = 0.569	
Substance type			
Drug	99 (60.0)	0.15 ± 0.18	(0.11-0.19)
Chemical	66 (40.0)	0.19 ± 0.24	(0.13-0.25)
		t = −1.308, p = 0.193	
Witnessed incident			
None	25 (15.2)	0.18 ± 0.22	(0.09-0.27)
Yes	140 (84.8)	0.17 ± 0.20	(0.14-0.20)
		t = −0.348, p = 0.728	
Home management			
None	120 (62.7)	0.15 ± 0.17	(0.12-0.18)
Yes	45 (27.3)	0.23 ± 0.27	(0.15-0.31)
		t = 2.051, p = 0.045*	
Arrival time to ED			
<1 hour	71 (43.0)	0.17 ± 0.22	(0.12-0.22)
1–2 hours	49 (29.7)	0.13 ± 0.20	(0.07-0.20)
>2 hours	45 (27.3)	0.21 ± 0.18	(0.16-0.26)
		F = 1.633, df = 2, p = 0.199	

Notes: *P-value: statistically significant at <0.05. t: student t-test, P: p-value. F: one way ANOVA, df: degree of freedom

Abbreviations: CI: confidence interval, PSS: poison severity score, BMI: body mass index

Two thirds of toddlers ingested various types of medications, while 40 % ingested various types of chemical products, Table 1. The most common medications were antipyretics & analgesics (n = 25), cardiac drugs (n = 10), and more than one type (n = 13). The forms of medications ingested varied between pills, capsules, creams, syrup and droplets. In chemical poisoning, 22 toddlers orally ingested sodium hydroxide (component of household product), while 14 ingested kerosene (petroleum product). Other ingested products are enlisted by their commercial names and generics, Table 2.

**Table 2:** Orally ingested substances

*Medication substance*	*n*	*Chemical substance*	*n*
Antipyretics/analgesics (Ibuprofen, diclofenac, celecoxib, aspirin, acetaminophen)	25	Hydrogen peroxide + Ammonium hydroxide (Hair dye)	5
Antidepressants (Mirtazapine, risperidone)	2	Chloroxylenol (Dettol)	2
Psychotics (Olanzapine, alprazolam)	3	Bleach (alkaline chemical) (Flash, bleach powder)	3
Neurological (Lisuride maleate, benzodiazepine, lamotrigine, valproic acid, carbamazepine)	6	Organophosphate (Insecticides, permethrin, naphthalene)	7
Hormone analogue (Levothyroxine, duphaston, desmopressin, cabergoline)	6	Paint thinner Rodenticide (Rat poison)	3 2
Gastrointestinal drugs (Pantozol, navidoxine, lorazepam)	4	Alcohol based chemical (Liquid perfumes, acetone)	4
Antibiotics (Norfloxacin, azithromycin, amoxicillin)	4	Surfactant (Fairy, Shampoo)	2
Creams (Gentian violet, diaper cream, sactol)	3	Petroleum product (Kerosene, benzene)	14
Vitamins/minerals (Vitamin D, cod liver oil, multivitamin pills, ferrous sulfate)	7	Sodium hydroxide + chlorine (Clorox)	22
Antihistamines (Pizotifen, ketotifen, chlorpheniramine maleate, Actifed, loratadine/pseudoephedrine)	9	Natural dye (Local herbal product)	1
Contraceptives (Yasmin, microgynon)	6	Unidentified chemical	1
Hypoglycemic (Glibenclamide)	1		
Cardiac drugs (Metoprolol, lisinopril, hydralazine, digoxin, lozartan, bisoprolol, atenolol, amlodipine)	10		
Multiple drugs (At least 2 or more of the above)	13		
Total	99	Total	66

Note: n= number of cases

Almost 85 % of poison incidents were witnessed by one of the parents. Rough estimates of poison amounts were reported by parents, thus this variable was not accounted statistically. Prior to ED arrival, some parents (27.3 %) provided various types of home remedies to their poisoned toddlers. Mutually exclusive home remedies included forcing the toddler to drink plain water (n = 17), lemon juice (n = 5), milk (n = 10), yogurt (n = 2) and salt/sugar solutes (n = 1). In addition, manually induced vomiting with or without fluid administration (n = 26) was reported. The time between the poison incident and arrival to ED ranged between 0.3-3.4 hours.

### Poison Severity Scores

**Fig. 2:**
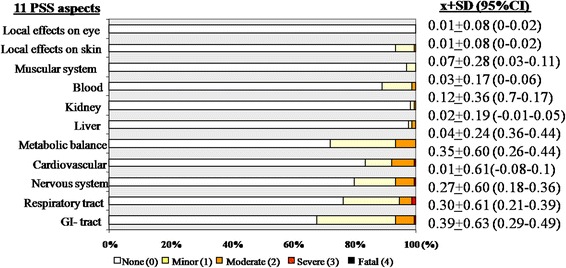
Frequency distribution and mean scores of the 11 Poison Severity Score aspects

Qualitative evaluation showed that the majority of toddlers were asymptomatic on all of the 11 PSS aspects (67-94 %), Fig. 2. No fatal cases were observed. The composite mean PSS among all poisoned toddlers was 0.16 ± 0.21 (95 % CI: 0.13-0.19), and it was the highest at the GI aspect 0.39 ± 0.63 (95 % CI: 0.29-0.49), followed by Metabolic balance aspect 0.35 ± 0.60 (95 % CI: 0.26-0.44) and others.

Bivariate analysis showed no statistically significant differences in the composite mean of PSS between the sex, age category, BMI, and substance type groups. The only significant difference was observed among toddlers receiving home remedies who complained of higher composite mean of PSS 0.23 ± 0.27 (0.15-0.31), p = 0.045, Table 2. A multilinear regression model was constructed to further investigate the combined effect of all exposures and to adjust for all possible confounders, Table 3. Higher composite mean of PSS was also significantly associated with toddlers who received home remedies (adj.p = 0.048) compared to those who directly visited ED. Individual aspects of PSS showed that chemical poisoning had significantly higher severity scores at the respiratory aspect (adj.p = 0.047) and the muscular aspect (adj.p = 0.009) compared to medication poisoning. Unwitnessed incidents was associated with higher severity on the muscular aspect (adj.p = 0.026). Delayed arrival times to the ED was significantly associated with higher severity scores at the GI aspect (adj.p = 0.001), nervous system aspect (adj.p = 0.014) and kidney aspect (adj.p < 0.001).

**Table 3:** Significantly associated factors with higher poison severity scores.

Exposures Outcomes	Gender Female:Male	Age (Years)	BMI (Kg/m^2^)	Substance type Drug:Chemical	Witnessed incident No : Yes	Home management No : Yes	Arrival time to ED (hours)
	Beta (t) *P-value*	Beta (t) *P-value*	Beta (t) *P-value*	Beta (t) *P-value*	Beta (t) *P-value*	Beta (t) *P-value*	Beta (t) *P-value*
Composite mean of PSS	*−0.014 (−0.15) P = 0.883*	*−0.049 (−0.51) P = 0.612*	*−0.047 (−0.49) P = 0.622*	*0.136 (1.38) P = 0.170*	*−0.071 (−0.74) P = 0.464*	0.189 (1.99) P = 0.048*	*0.189 (1.96) P = 0.052*
GI- tract	*−0.097 (−1.04) P = 0.301*	*−0.050 (−0.53) P = 0.597*	*−0.106 (−1.13) P = 0.262*	*0.069 (0.71) P = 0.480*	*0.017 (0.18) P = 0.861*	*0.062 (0.66) P = 0.508*	0.314 (3.31) P = 0.001*
Respiratory tract	*0.126 (1.34) P = 0.185*	*−0.097 (−1.01) P = 0.315*	*−0.001 (−0.01) P = 0.990*	0.195 (2.01) P = 0.047*	*−0.08 (−0.84) P = 0.401*	*0.165 (1.76) P = 0.081*	*−0.068 (−0.71) P = 0.477*
Nervous system	*0.051 (0.53) P = 0.597*	*0.039 (0.40) P = 0.689*	*−0.122 (−1.26) P = 0.211*	*0.026 (0.26) P = 0.793*	*−0.032 (−0.33) P = 0.740*	*0.049 (0.51) P = 0.610*	0.242 (2.49) P = 0.014*
Cardiovascular	*−0.138 (−1.46) P = 0.149*	*−0.003 (−0.04) P = 0.972*	*−0.003 (−0.03) P = 0.975*	*−0.008 (−0.08) P = 0.933*	*−0.143 (−1.49) P = 0.140*	*0.176 (1.87) P = 0.064*	*−0.117 (−1.22) P = 0.227*
Metabolic balance	*0.118 (1.22) P = 0.226*	*0.001 (−0.003) P = 0.998*	*0.044 (0.45) P = 0.651*	*0.107 (1.08) P = 0.285*	*0.004 (0.04) P = 0.969*	*0.075 (0.78) P = 0.438*	*0.127 (1.29) P = 0.197*
Liver	*−0.078 (−0.79) P = 0.430*	*−0.051 (−0.52) P = 0.608*	*0.002 (0.02) P = 0.985*	*0.067 (0.67) P = 0.507*	*0.094 (0.95) P = 0.345*	*−0.058 (−0.59) P = 0.554*	*−0.062 (−0.62) P = 0.535*
Kidney	*−0.050 (−0.57) P = 0.572*	*−0.111 (−1.24) P = 0.219*	*0.123 (1.37) P = 0.173*	*0.103 (1.13) P = 0.262*	*0.040 (0.44) P = 0.662*	*0.174 (1.97) P = 0.051*	0.353 (3.94) P < 0.001*
Blood	*−0.079 (−0.81) P = 0.417*	*0.028 (0.29) P = 0.775*	*0.019 (0.19) P = 0.843*	*−0.121 (−1.21) P = 0.231*	*−0.038 (−0.39) P = 0.700*	*0.003 (0.03) P = 0.975*	*0.119 (1.21) P = 0.228*
Muscular system	*−0.053 (−0.57) P = 0.569*	*−0.008 (−0.08) P = 0.934*	*−0.029 (−0.31) P = 0.755*	0.255 (2.65) P = 0.009*	−0.215 (−2.26) P = 0.026*	*0.088 (0.94) P = 0.348*	*0.084 (0.89) P = 0.378*
Effects on skin	*−0.015 (−0.16) P = 0.876*	*−0.036 (−0.36) P = 0.720*	*−0.101 (−1.02) P = 0.310*	*0.072 (0.710) P = 0.479*	*0.027 (0.27) P = 0.788*	*0.135 (1.39) P = 0.168*	*−0.034 (−0.35) P = 0.729*
Effects on eye	*0.065 (0.68) P = 0.501*	*−0.049 (−0.50) P = 0.618*	*0.036 (0.37) P = 0.713*	*0.133 (1.35) P = 0.181*	*0.031 (0.313) P = 0.755*	*0.186 (1.95) P = 0.054*	*−0.059 (−0.61) P = 0.545*

Note: *Statistically significant at p < 0.05

Abbreviations: Beta, coefficient of determination; t = student t-test; Kg: kilogram; m:meter, P, p-value

## Discussion

Almost 67 % of toddlers in this study were asymptomatic regarding at least one of the 11 PSS aspects. This was comparable to the Ireland 2013 poison center that reported 70 % of their patients to be asymptomatic [24]. Authors suspected that adopting the composite mean score of the 11-item PSS might not be accurately reflecting the actual mortality risks and severity of poisoning incident. Upon testing the 11 PSS aspects as individual outcome parameters, a number of associated factors leading to higher severity scores emerged. Chemical poisoning in this study was significantly associated with higher severity scores at the nervous and muscular aspects. This was in alignment with what the literature has stated that respiratory distress is associated with numerous poisonous agents, most of which were chemical products [25]. Abnormal muscular aspects such as pain or tenderness are common with the ingestion of chemical products due to their erosive nature [18]. ED physicians can anticipate for abnormal clinical outcomes and initiate early medical interventions, simply by identifying the nature of the substance ingested upon arrival.

Higher poison severity scores were associated with home remedies. This rejects the null hypothesis that home remedies (as perceived by parents) would improve the clinical outcomes. Authors speculated that the presence of other confounders (mainly substance type or amount) might have influenced the initial bivariate analysis, but even after regression home remedies remained statistically associated with higher severity scores (adj.p = 0.048). The two main types of home remedies reported by parents in this study were orally administered fluids and/or manually induced vomiting. Food and beverages can have a profound impact on prescribed medications as they are known to increase, neutralize, or cease their desired effect [26–30] . This fact might apply to the pharmacological doses of medications, but not to the toxicological doses in poisoning, thus the administration of foods or fluids might not be effective at all. One study recommended drinking milk or water if a corrosive chemical product is orally ingested [31]. In this study, parents decided that such fluids might minimize the effects of the substance ingested without realizing that it all depends on the chemical nature of the substance itself. Manually induced vomiting was a risky and unpleasant practice that exerted physical and psychological stress on the toddler. It is usually associated with a number of unwanted acute complications such as electrolyte/fluid imbalances and aspirations [32]. Things can become even worse in case the chemical substance ingested was irritating since it may damage the lining of the esophagus, pharynx and oral mucosal surface during vomiting [18]. Induced vomiting by physically stimulating a gag reflex might harm or cause death as reported by a study [33].

Delayed ED arrival times was significantly associated with higher severity scores at the GI, nervous system, and kidney aspects. The time between ingestion and ED was reported to have a great effect on the efficiency of ED treatments especially poison antidotes, thus compromising the clinical outcomes [21]. Moreover, a study found that a delayed arrival time beyond 3 hours, significantly increased the hospital length of stay [34]. Delayed time is highly dependent on the issue of witnessing the poison incident. In this study, unwitnessed incidents (15.2 %), was significantly associated with an increased severity score at the muscular aspect. Studies recommended an immediate ED admission in case of a suspected poison incident has occurred [21, 35].

Sample characteristics in this study were consistent with those reported in several studies [36–38]. National estimates of incident cases and population based poisoning rates sourced from 100 EDs within the USA announced that 72.3 % of incidents were committed by toddlers making them indeed the highest risk group among children [36, 37]. In this study, poisoned toddlers estimated to 226/315 (71.7 %) over a 2 year period. However, the Spanish society of pediatric emergencies stated that there is sex differences among poisoned children [15], unlike findings in this study. Oral route of poising was the most common during the 2 year period 300/315 (95.2 %) which was also similar to literature findings [38].

### Limitations

This study has generated analysis from a single poison center on a relatively small sample size, which might limit its generalization to other settings. The time limit of data collection could have been extended further to recruit more eligible cases of poisoning. However, study investigators had to abide with the approved time limit of data collection. Authors admit that there is no true denominator as some poisoned toddlers might have recovered at home and were never presented to the ED.

Amount of poison was not accounted statistically as a potential contributing factor due to the diverse nature and forms of the substances ingested (powder, cream, fluid, pills, capsules, etc.), besides the fact that it has been reported by parents in rough estimates. The incident details were reported by parents under stress, thus a recall and/or cognitive bias was suspected during the initial ED visit. This was overcome by phone contacting the parent at a later time to revalidate the reported data.

## Conclusions

Home remedies were significantly associated with higher severity scores among poisoned toddlers, a high-risk age group for orally ingested substances. Chemical poison ingestions were associated with higher respiratory and muscular poison severity scores compared to medication poisoning. Delayed arrival times to the hospital after the poisoning incident was significantly associated with higher GI, nervous system and kidney poison severity scores.

### Recommendations

Study investigators recommend parents to adhere with the local and international poison management guidelines. Parents need to be informed through community awareness campaigns that the initial response to any suspected or witnessed substance ingestion is notifying a nearby poison center. The launching of a unified hotline poison control number in Saudi Arabia is essential and it’s placement at homes will definitely cut-off delays in the arrival time to ED. Due to the fact that poison home remedies do exist in the community and testing it in randomized control trials is not scientifically and ethically applicable, poison centers need to inquire further on such data from parents who commit such practices. Therefore, it is advisable to incorporate it within the Saudi MOH and NDPIC reporting forms for drug over dosage or chemical poisoning, to further investigate the spread and outcomes of such practices.

### Availability of Data and Materials

Data sets of this study are available in SPSS formats with the corresponding author (please refer to corresponding address). In addition, hardcopies of consents and data collection forms will be stored for 2 years after publication before being properly disposed.

## Abbreviations

AAP: American Academy of Pediatrics
AAPCC: American Association of Poison Control Centers
ED: Emergency department
KAMC: King Abdulaziz Medical City
JCI: Joint Commission International
MNG-HA: Ministry of National Guard Health Affairs
NDPIC: National Drug & Poison Information Center
SFDA: Saudi Food and Drug Authority
KAIMRC: King Abdullah International Medical Research Center
MOH: Ministry of Health
MRN: Medical record number
BMI: Body mass index
adj.P: Adjusted P value
adj.OR: Adjusted odds ratio
CI: Confidence interval
